# Green biosynthesis of bimetallic selenium–gold nanoparticles using *Pluchea indica* leaves and their biological applications

**DOI:** 10.3389/fbioe.2023.1294170

**Published:** 2024-01-11

**Authors:** Ahmed Mohamed Aly Khalil, Ebrahim Saied, Alsayed E. Mekky, Ahmed M. Saleh, Omar Mahmoud Al Zoubi, Amr H. Hashem

**Affiliations:** ^1^ Biology Department, Faculty of Science Yanbu, Taibah University, Medina, Saudi Arabia; ^2^ Department of Botany and Microbiology, Faculty of Science, Al-Azhar University, Cairo, Egypt

**Keywords:** anticancer activity, antimicrobial activity, bimetallic nanoparticles, leaf extract, phytochemical analysis, *Pluchea indica*

## Abstract

Increasing bacterial resistance and the negative impact of currently used antibacterial agents have produced the need for novel antibacterial agents and anticancer drugs. In this regard, nanotechnology could provide safer and more efficient therapeutic agents. The main methods for nanoparticle production are chemical and physical approaches that are often costly and environmentally unsafe. In the current study, *Pluchea indica* leaf extract was used for the biosynthesis of bimetallic selenium–gold nanoparticles (Se-Au BNPs) for the first time. Phytochemical examinations revealed that *P. indica* leaf extract includes 90.25 mg/g dry weight (DW) phenolics, 275.53 mg/g DW flavonoids, and 26.45 mg/g DW tannins. X-ray diffraction (XRD), transmission electron microscopy (TEM), Fourier-transform infrared (FTIR) spectroscopy, dynamic light scattering (DLS), scanning electron microscopy (SEM), and energy-dispersive X-ray spectroscopy (EDX) techniques were employed to characterize Se-Au BNPs. Based on UV-vis spectra, the absorbance of Se-Au BNPs peaked at 238 and 374 nm. In SEM imaging, Se-Au BNPs emerged as bright particles, and both Au and Se were uniformly distributed throughout the *P. indica* leaf extract. XRD analysis revealed that the average size of Se-Au BNPs was 45.97 nm. The Se-Au BNPs showed antibacterial properties against *Escherichia coli*, *Pseudomonas aeruginosa*, *Staphylococcus aureus*, and *Bacillus subtilis*, with minimum inhibitory concentrations (MICs) of 31.25, 15.62, 31.25, and 3.9 μg/mL, respectively. Surprisingly, a cytotoxicity assay revealed that the IC_50_ value toward the Wi 38 normal cell line was 116.8 μg/mL, implying that all of the MICs described above could be used safely. More importantly, Se-Au BNPs have shown higher anticancer efficacy against human breast cancer cells (MCF7), with an IC_50_ value of 13.77 μg/mL. In conclusion, this paper is the first to provide data on the effective utilization of *P. indica* leaf extract in the biosynthesis of biologically active Se-Au BNPs.

## 1 Introduction

Antimicrobial resistance, the ability of a microorganism to resist various antimicrobial agents, is a major threat affecting human health worldwide (Prestinaci et al., 2015). In this specific manifestation of resistance, bacteria develop the capacity to counteract the impact of previously efficacious medicine (Colson et al., 2021). In certain instances, the microorganism acquires the ability to withstand the effects of numerous medications, a condition commonly referred to as multidrug resistance (MDR). Antimicrobial resistance can arise as a result of numerous causes, such as genetic modifications, acquired resistance from other species, and inherent resistance displayed by some microbes against specific antimicrobial agents (Catalano et al., 2022). Indeed, the abuse of antibiotics is a major cause of the evolution of multidrug-resistant bacteria (Ventola, 2015).

Cancer is well recognized as a persistent, diverse ailment that originates at the genetic, phenotypic, and pathological levels and advances via various clinical presentations (Dheyab et al., 2022; Alafaleq et al., 2023). Cancer, with its elevated prevalence, has emerged as a prominent global health issue in the 21st century, ranking as the second leading cause of mortality on a global scale. Each year, approximately 15 million fatalities occur as a result of the continued presence of cancerous cells, and this figure is consistently increasing (Tabrez et al., 2022a). Breast cancer is the most common cancer diagnosed and the leading cause of cancer-related deaths among women worldwide. Although there is a large selection of chemotherapeutic agents available for the treatment of cancer, it is crucial to remember that these drugs can have serious side effects on several different human organs (Schirrmacher, 2019). Thus, many scientists have suggested safer alternatives to antimicrobial and anticancer drugs. In the last few decades, chemists working in green chemistry have worked extremely hard to create less dangerous synthetic processes (Abdussalam-Mohammed et al., 2020). In this context, nanobiotechnology has typically attracted much attention in relation to efforts to boost the exploitation of nano-sized materials in advanced biotechnology (Dash et al., 2020). Nanomaterials are useful in various fields, including environmental science, electronics, agriculture, and biomedicine (Ali et al., 2022). Nanomedicine is the application of precisely engineered nanomaterials to the development of new human therapeutic and diagnostic modalities. The intersection of medicine and nanotechnology has created new opportunities in therapeutic and pharmaceutical research (Khan et al., 2021; Hashem et al., 2022a; Hasanin et al., 2023).

A wide range of physical and chemical methods have been employed to produce nanomaterials, including sonochemical methods, microwaves, electrochemical methods, sol–gel, hydrothermal processes, co-precipitation, chemical vapor deposition, and polyol synthesis (Huston et al., 2021; Saied et al., 2022a; Tabrez et al., 2022b). Unfortunately, these methods have environmental issues, such as the use of hazardous chemicals, are labor-intensive. and require specialized instruments and vacuum settings (Hachem et al., 2022; Solanki, 2022; Suroshe, 2023). In ultrasonic baths, it is extremely hard to control the temperature. This is because ultrasonic baths usually warm up when used, resulting in an inconsistent temperature. Many sonochemical reactions must be carried out at a certain temperature, which is a major disadvantage. Furthermore, sonochemical methods have low efficiency, this is an expensive technique, and control of the deposition parameters is difficult to achieve (Modan, 2020; Dheyab et al., 2021b). In this regard, the use of green synthesis of nanomaterials could provide a worthwhile ecofriendly approach (Abdelaziz et al., 2022; Hashem and Salem, 2022; Salem et al., 2022; Zughaibi et al., 2023). The use of plant extracts as biological materials under moderate conditions is a part of these green procedures (Shafey, 2020; Ceylan et al., 2021). Flavonoids, alkaloids, phenols, terpenoids, and quinines are beneficial phytochemicals that contribute significantly to the creation of nanomaterials with precise control over size and distribution (Iashin et al., 2021). Additionally, by serving as reducing and capping agents, organic natural ingredients make it easier to create nanomaterials (Dikshit et al., 2021; Shreyash et al., 2021; Tabrez et al., 2022c).

Gold nanoparticles (AuNPs) have been widely used in photothermal treatment, photoacoustic imaging, and targeted drug delivery (Siddique and Chow, 2020). AuNPs have attracted increasing attention due to their exceptional and unique physicochemical properties, which are relevant in a range of applications in material science, biomedicine, catalysis, and quantum dot technology. These features include electrical conductivity, low toxicity, a high absorption spectrum, photothermal effects, adjustable size- and shape-dependent optical properties, biocompatibility, chemical stability, and easy functionality (Ali Dheyab et al., 2020; Ali Dheyab et al., 2021b). For several years, AuNPs have been the focus of extensive scientific and technological research (Hu et al., 2020; Ali Dheyab et al., 2021a; Sarfraz and Khan, 2021). Au is typically an inert or non-reactive metal, but at the nanoscale, its electron behavior drastically changes, completely changing its properties (Dheyab et al., 2021a; Dheyab et al., 2021c). With biocompatibility and minimal cytotoxicity advantages, AuNPs have emerged as a successful drug-delivery system in human cancer and cell biology (Chandrakala et al., 2022; Dheyab et al., 2023a). Biosynthesized AuNPs can potentially be used as nanomedicines to eradicate breast cancer cells by utilizing mushroom extract via a sonochemical process for 5 min. The strong antibacterial activity of gold nanoparticles is well known, especially against Gram-negative bacteria. They are also well known for being highly stable, having little cytotoxicity, and being simple to synthesize. Unlike other nanoparticles, AuNPs provide easy surface modification, which facilitates the creation of novel antibacterial combinations (Dheyab et al., 2023c). Au nanospheres, Au nanocages, Au nanoshells, and Au nanorods are the four main types of AuNPs reported by Dheyab et al. (2023b). They also discuss the advantages and disadvantages of each type of AuNP in PTT. Dheyab et al. (2020) synthesized gold-coated Fe_3_O_4_ (Au–Fe_3_O_4_ NPs) for use in photothermal therapy to kill MCF-7 breast cancer cells. Using an extract of the fresh mature fruits of the Iraqi *Prosopis farcta* plant (Fabaceae), Rabeea et al. (2021) created AuNPs and examined their catalytic activity.

The use of selenium in nanoparticles (SeNPs) has been suggested to be effective in anticancer therapy (Afshari et al., 2022). Despite the outstanding anti-tumor activity of SeNPs, their low stability and ease of aggregation restrict their utilization (Li et al., 2023). Accordingly, a method to get around the limitations of working with single-metal nanoparticles is to combine two metal NPs with different structural characteristics to create bimetallic nanoparticles (BNPs) (Rajeev et al., 2021). The distinctive geometrical structure and mixing pattern of bimetallic nanoparticles enhance their use (Behera et al., 2020). Regarding activity and durability, BNPs have proven to be more effective than their monometallic counterparts in various aspects, and they achieve this without the need for expensive or specialized equipment (Ghosh et al., 2023). Furthermore, green approaches to the synthesis of BNPs bring added value as they are environmentally safe. The efficiency of a coastal blooming plant of the Asteraceae family, *Pluchea indica*, in the green biosynthesis of NPs has been reported. However, its potential for biosynthesizing BNPs is yet to be investigated (Chiangnoon et al., 2022; Al-Askar et al., 2023; Bi et al., 2023). This study used the leaf extract of *P. indica* to biosynthesize Se–Au BNPs. The Se–Au BNPs produced were fully characterized using UV-vis, scanning electron microscopy (SEM), transmission electron microscopy (TEM), Fourier-transform infrared (FTIR) spectroscopy, and X-ray diffraction (XRD), and their bioactivity (antibacterial and anticancer) was investigated.

## 2 Materials and methods

### 2.1 Preparation of *P. indica* leaf extract

*P. indica* leaves were collected from Ayyat city, Giza Governorate, Egypt. After careful cleaning with double-distilled water, the gathered leaves were allowed to dry for 5 days to remove impurities. A measure of 100 mL of deionized water was combined with 5 g of the chopped material to create the extract. The sample was then heated to a temperature of 65 °C for 60 min, after which it was separated by pouring out the liquid. The supernatant underwent centrifugation at a speed of 10,000 revolutions per minute for 10 min. Subsequently, it was stored at a temperature of 4 °C for 1 week to ensure its usability (Molaei et al., 2015).

### 2.2 Qualitative screening and quantitative determination of phytochemicals in *P. indica* extract

Qualitative phytochemical screening is often used to determine whether phytochemicals are present or absent in plant crude extract. The results are given as either present (positive [+]) or absent (negative [-]). Molisch’s test for the presence or absence of carbohydrates was conducted according to Sofowora (1993). Frothing tests were performed for saponins according to the ferric chloride test, and for tannins according to Mohite et al. (2020). The Biuret test for proteins and amino acids was conducted according to Salna et al. (2011). Lead acetate, NaOH, aluminum chloride, and H_2_SO_4_ tests were conducted for flavonoids (Win et al., 2019). Following Prashant et al. (2011), the ferric chloride test was used for phenolics. A glycoside test and Bontrager’s test for glycosides were conducted, and the Keller–Kiliani and Legal’s tests were conducted for cardiac glycosides (Prashant et al., 2011). Wagner’s test, Dragendorff’s test, and Hager’s test for alkaloids were conducted according to Prashant et al. (2011). The HCl test for phlobatannins was conducted. The alcoholic potassium hydroxide test and ninhydrin test were conducted for quinone. Total phenolics, flavonoids, and tannins were all determined quantitatively. Total phenolic content (TPC) was determined by using the Folin–Ciocalteu technique, as described by Makkar (2003), to determine the quantity of total phenolics in the sample. Total flavonoid content (TFC), a measure used by Shraim et al. (2021), was calculated to determine the quantity of flavonoids contained in the sample. Total tannins were estimated by using the Folin–Ciocalteu technique, as described by Makkar (2003), to determine the total quantity of tannins present in the sample.

### 2.3 Biosynthesis of Se–Au BNPs using *P. indica* extract

Solutions of Na_2_SeO_3_ (1 mM) and HAuCl_4_⋅3H_2_O (1 mM) were prepared. The reaction mixture employed in the experiment was prepared as follows: the liquid underwent rapid agitation at a temperature of 40°C after addition of 30 mL of Na_2_SeO_3_, 30 mL of HAuCl_4_⋅3H_2_O, and 40 mL of the plant extract. In order to decrease the pH to 8.0, aliquots of 1 N NaOH were introduced into the solution, which was then subjected to agitation at a temperature of 40 °C for 1 h. The mixture was then allowed to sit at room temperature for the next day (Cittrarasu et al., 2021). Using a UV-vis spectrophotometer, it was observed that the mixture hue changed to a deep violet tint, suggesting the synthesis of Se–Au BNPs. Centrifugation was employed to achieve the separation of the precipitate. Subsequently, the separated precipitate underwent a thorough washing procedure, involving three cycles of rinsing with deionized water. Finally, the precipitate was dried in an oven, where it was exposed to a temperature of 200 °C for 3 h (Thangamani and Bhuvaneshwari, 2019).

### 2.4 Characterization of Se–Au BNPs

The initial step was the assessment of the biogenic Se–Au BNPs using a UV-vis spectrophotometer (Jenway-6305, Staffordshire, United Kingdom) within a wavelength range of 200–800 nm. The average particle size distribution of the generated Se–Au BNPs was determined by dynamic light scattering (Nano ZS, Malvern, United Kingdom). In addition, we utilized a TEM, model JEM-1230 (manufactured in Japan, specifically in Akishima, Tokyo 196-8558), to examine the average and precise dimensions and morphology of the synthesized Se–Au BNPs. Furthermore, we adopted the selected area electron diffraction technique for this purpose (Hwang et al., 2020). The XRD technique, specifically employing an XRD-6000 instrument (Shimadzu Scientific Instruments, Japan), was utilized to ascertain the crystal size and crystallinity of the Se–Au BNPs. The surface morphology and boundary size of the synthesized Se–Au BNPs were analyzed using a scanning electron microscope coupled to an energy-dispersive X-ray (SEM-EDX) unit. The SEM used for this analysis was a ZEISS EVO-MA10 microscope (Germany), while the EDX unit was provided by BRUKER (Germany). This analysis aimed to examine the elemental arrangement, purity, and dispersal of the components in the synthesized Se–Au BNPs. Subsequently, FTIR analysis was conducted to ascertain the chemical functional groups established between the synthesized selenium–gold bimetallic nanoparticles and the extract derived from the *P. indica* leaves. The FTIR analysis was carried out using a JASCO FT-IR 3600 instrument, employing the KBr pellet approach, with a wavenumber ranging from 400 to 4,000 cm^-1^.

### 2.5 Antimicrobial activity

The antimicrobial activity of Se–Au BNPs, the starting materials (Na_2_SeO_3_ and HAuCl_4_⋅3H_2_O), and the standard antibiotic (norfloxacin, Nor) against *Escherichia coli* ATCC 25922, *Pseudomonas aeruginosa* ATCC 27853, *Bacillus subtilis* ATCC 6051, and *Staphylococcus aureus* ATCC 25922 was evaluated using the agar well diffusion technique. The agar diffusion test was conducted in accordance with the guidelines indicated in the M51-A2 document of the Clinical Laboratory Standard Institute (Standards, 2002), with several changes. The bacterial and fungal strains examined were streaked separately onto Mueller–Hinton agar and malt extract agar plates. Agar wells were created using a sterile cork borer with a diameter of 8 mm. Subsequently, 100 µL of Se–Au BNPs, Na_2_SeO_3_, HAuCl_4_⋅3H_2_O, and the standard antibiotic at concentrations of 500 μg/mL were introduced into the wells. The samples were then incubated at 37°C for 24 h. Subsequently, the width of the inhibition zone was measured for each treatment. In order to ascertain the minimal inhibitory concentration, several concentrations of both Se–Au BNPs and Nor were prepared and assessed for their antimicrobial efficacy (Vargas et al., 2007; Dacrory et al., 2021; Hashem et al., 2021).

### 2.6 *In vitro* cytotoxicity and anticancer activity

The MTT test was employed to assess the cytotoxicity of biosynthesized Se-Au BNPs against both a normal cell line (Wi38 ATCC CCL-75) and a malignant cell line (MCF7 ATCC HTB-22) (Van de Loosdrecht et al., 1994; Khalil et al., 2021). A tissue culture plate (96 wells) was inoculated with 1 × 10^5^ cells/mL (100 µL/well) and incubated at 37°C for 24 h to develop a complete monolayer sheet. Growth medium was decanted from 96-well microtiter plates after formation of a confluent sheet of cells, and the cell monolayer was washed twice with wash media. The tested sample was diluted two-fold in RPMI medium with 2% serum (maintenance medium). Subsequently, 0.1 mL of each dilution was tested in different wells, leaving three wells as controls containing only maintenance medium. The plate was then incubated at 37°C and examined. The cells were checked for any physical signs of toxicity, e.g., partial or complete loss of the monolayer, rounding, shrinkage, or cell granulation. MTT solution was prepared (5 mg/mL in PBS) (Bio Basic Inc., Canada) and 20 µL of MTT solution was added to each well; the samples were then shaken at 150 rpm for 5 min, followed by incubation at 37°C with 5% CO_2_ for 4 h to allow the MTT to be metabolized. The optical density was measured at 560 nm. Cell viability and cell inhibition percentages were calculated according to Eqs 1 and 2.

$$Viability\;\%=\frac{Test\;OD}{Control\;OD}\;X\;100,$$ (1)

Inhibition %=100−Viability %. (2)

## 3 Results and discussion

### 3.1 Phytochemical analyses of leaf extract of *P. indica*

Qualitative phytochemical screening of *P. indica* leaf extract was conducted in this study (Table 1). The water extract of *P. indica* leaves contained carbohydrates, phenolics, tannins, flavonoids, and glycosides. On the other hand, phlobatannins, saponins, alkaloids, quinone, cardiac glycosides, sterols, and terpenes were not observed.

**TABLE 1:** Preliminary phytochemical screening of *P. indica* leaf extract.

Phytochemical		Test	Leaf extract of *P. indica*
1	Carbohydrates	Molisch’s test	+ve
2	Fixed oils and fats	Saponification test	-ve
3	Phenolics	Ferric chloride test	+ve
4	Tannins	Ferric chloride test	+ve
5	Phlobatannins	HCL test	-ve
6	Flavonoids	Lead acetate test	+ve
		AlCL_3_ test	+ve
7	Saponins	Froth test	-ve
8	Glycosides	Glycoside test	+ve
		Conc. H_2_SO_4_ test	+ve
9	Alkaloids	Dragendorff’s test	-ve
		Wagner’s test	-ve
		Hager’s test	-ve
10	Quinone	KOH test	-ve
11	Sterols and terpenes	Salkowski’s test	-ve
12	Cardiac glycosides	Legal’s test	-ve
		Keller–Kiliani test	-ve

+ve = present; -ve = absent.

Moreover, quantitative analyses were carried out to determine the levels of total flavonoids, phenolics, and tannins in the leaf extract of *P. indica*, as shown in Table 2. The results showed that total flavonoid levels were lower than total levels of phenolics and tannins; the quantity was 90.25 ± 1.04 mg rutin/g DW. Flavonoids are a diverse class of polyphenolic chemicals characterized by a benzoyl-γ-pyrone structure, and they are widely distributed across the plant kingdom (Jalil et al., 2022). Furthermore, the total amounts of phenolics and tannins in the leaf extract of *P. indica* were 275.53 ± 2.39 GA E/g DW and 26.45 ± 0.67 mg TA/g DW, respectively. The term “tannin” is frequently used to refer to a complex polyphenolic biomolecule that possesses several hydroxyl and other functional groups, such as carboxyl, that enable it to form robust complexes with other macromolecules (Baldwin and Booth, 2022). Phenolic compounds are created through shikimic acid and phenylpropanoid pathways (Lin et al., 2016). In accordance with the present results, *P. indica* is regarded as a rich source of phenolics and flavonoids. For instance, the presence of 124.8 g total phenols and 46.3 g flavonoids/g dried *P. indica* leaves extracted in 20% methanol has been recorded (Mahasuari et al., 2020). Cho et al. (2012) reported the presence of phenolics and flavonoids (78.9 and 40.4 mg/g DW, respectively), but not tannins, in the aqueous extract of *P. indica* roots.

**TABLE 2:** Determination of total phenolics, flavonoids, and tannins in the leaf extract of *P. indica*.

Active compound	Leaf extract of *P. indica*
Total phenolics (mg GAE/g DW)	275.53 ± 2.39^a^
Total flavonoids (mg rutin/g DW)	90.25 ± 1.04^b^
Total tannins (mg TA/g DW)	26.45 ± 0.67^c^

Data are presented in the form mean ± SE for three replicates (n = 3).

### 3.2 Biosynthesis of Se–Au BNPs using *P. indica* leaf extract

The synthesis of the NPs required biomolecules known as capping and reduction agents, which are present in the filtrate of *P. indica* extract (Rana et al., 2023). A capping agent is crucial in controlling size and form with low-tech, low-cost, and energy-efficient tools and procedures (Brar et al., 2022). These biomolecules also act as a backup method of creating a monolayer on the surface of the NPs in order to prevent agglomeration (Hashem et al., 2023). Following the formation of Se–Au BNPs, the color of the solution changed from a clear ruby pink to a grayish red color, signifying the total reduction of metal ions. An increase in metal ion reduction occurs when different biomolecules found in plant extracts are presented (Sardar and Mazumder, 2019). The surface functionalization of nanoparticles with biomolecules has been confirmed in recent investigations of biological extract-mediated NP production, which also increases their bactericidal action (Alagesan and Venugopal, 2019; Al-Radadi, 2021). Gold and silver nanoparticles were created by laser ablation in a chitosan–PVA mix solution by Awwad et al. (2021). According to Elemike et al. (2019a) and Elemike et al. (2019b), the various aqueous extracts of *Solidago canadensis* and *Stigmaphyllon ovatum* have been used to create the Ag–Au alloy. Emam (2019) stated that Arabic gum has been used as a biosynthesizer to create nanoalloy Ag–Au BMNPs. In a study by dos Santos Souza et al. (2022), SeNPs were synthesized using extracts derived from three distinct plant species, namely, *Allium cepa* (commonly known as onion), *Malpighia emarginata* (also referred to as acerola), and *Gymnanthemum amygdalinum* (commonly known as boldo). Zarharan et al. (2023) prepared a nanocomposite containing selenium–gold–chitosan (Se–Au–CS) as an anti-angiogenesis and antioxidant material. Selenium nanoparticles (Se-NPs) have been produced by Hashem et al. (2023) using pomegranate peel extract (PPE). Mirzaei et al. (2021) used an aqueous extract of *Gracilaria corticata* to fabricate bimetallic nanoparticles (zinc–selenium) physically. Additionally, pomegranate (*Punica granatum*) peel extracts have been used as reducing agents for the biosynthesis of AuNPs (Patel et al., 2019). In addition, they have been used to create Au and Ag nanoparticles on bacterial nanocellulose film by inducing *in situ* deposition using *P. granatum* peel extract (Deshmukh et al., 2022).

### 3.3 Characterization of Se–Au BNPs

#### 3.3.1 Powder XRD

**FIGURE 1:**
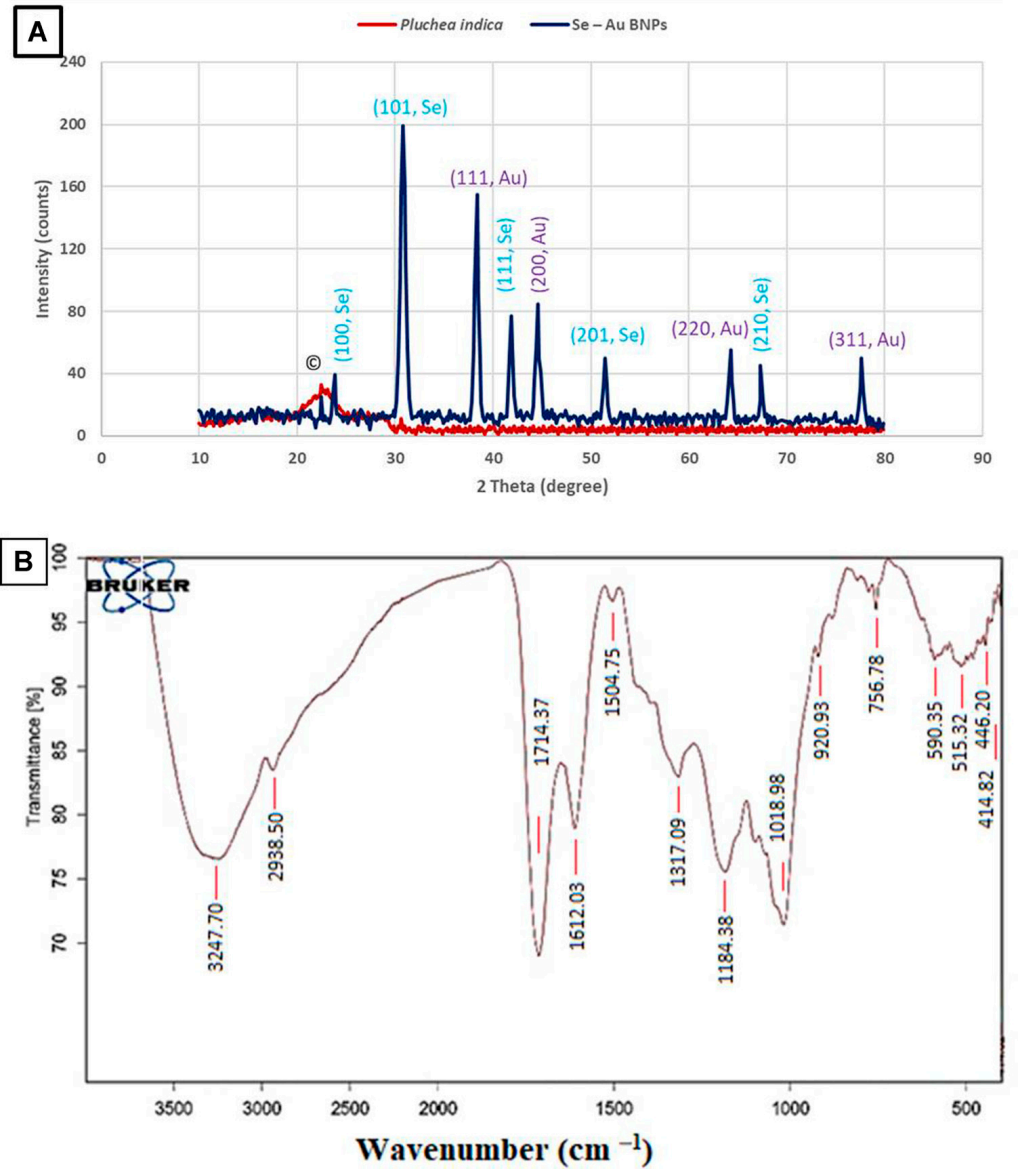
**(A)** XRD patterns of Se–Au BNPs and *P. indica* extract. **(B)** FTIR analysis of Se–Au BNPs.

XRD was utilized to analyze the structural properties of the Se–Au BNPs (Figure 1). A wide peak in the BNPs, ranging from 10^o^ to 80^o^ degrees, could be observed. Metallic particles exhibit unique patterns according to their microscopically determined structure. The XRD pattern of the synthetic Se–Au BNPs is shown in Figure 1A. The XRD analysis results supported the creation of nanocomplex Se–Au BNPs. Figure 1A shows the XRD diffraction peaks of the AuNPs; four peaks were observed. The obtained peak pattern was cross-referenced with the Joint Committee on Powder Diffraction Standards (JCPDS) file no. 65–8,601, which contains information about the crystalline structure of pure gold, as a point of comparison. The diffraction peaks observed at 2θ values of 38.39°, 44.57°, 64.29°, and 77.60° in the face-centered cubic (FCC) structure correspond to the (111), (200), (220), and (311) planes of the structure, respectively (Patil et al., 2019). The crystal structure and phase of the bimetallic Se–Au BNPs were also analyzed by XRD. In addition, Figure 1A displays the selenium nanoparticle XRD diffraction peaks, including peaks at 2θ of the 23.82° (100), 30.8° (101), 41.84° (111), 51.44° (201), and 67.34° (210) conventional selenium nanoparticle crystal planes (Santhosh et al., 2022), demonstrating the cubic crystalline structure of the synthesized Se–Au BNPs. Only one undeveloped peak related to *P. indica* research was observed, at 22.42° (denoted as ©); this research is focused on the production and longevity of Se–Au BNPs. The experimental and reference values demonstrated good agreement (Jaiswal et al., 2021; Qais et al., 2021). The produced Se–AuBNPs were firmly crystalline and connected with the amorphous *P. indica* extract, which promoted their diffusion in the solution for improved biomedical usage (Albalawi et al., 2022). The Debye–Scherrer equation was used to determine the mean size of the Se–Au BNP crystallites, which was determined to be 45.97 nm.

#### 3.3.2 FTIR analysis

FTIR analysis was utilized to identify the functional groups present in the composition of Se–Au BNPs, as depicted in Figure 1B. The solutions of Se–Au BNPs that were produced were dried in an oven set at 70 °C overnight in order to facilitate FTIR analysis. Subsequently, KBr pellets were used to prepare the sample. The Se–Au BNPs showed a characteristic band at 3,247 cm^−1^ associated with hydroxyl stretching. At 2,938 cm^─1^, the C=C stretching band of the alkyne group was observed. The presence of ketones, aldehydes, and carboxylic acids was indicated by a sharp peak at 1,714 cm^─1^, which was caused by the carbonyl group C = O. A brief peak at 1,612 cm^─1^ indicated the presence of unsaturated combinations (alkenes), while bands at 1,504 cm^─1^ indicated the deformation of ethyl methyl ether. A peak at 1,184 cm^─1^ (ethanoic anhydride stretching) showed the presence of ethers and esters, whereas the band at 1,317 cm^─1^ (CH_2_ bending) was related to the occurrence of cellulose. The band at 1,018 cm^─1^ was attributed to vibrations of C=O=C (Srivastava et al., 2021; Hashem et al., 2022b; Yarian et al., 2023). The *P. indica* spectrum was identical to that found in the literature review, as seen in Figure 1B, which shows a peak at 756 cm^1^ for -CCH and -COH bending (Lidiawati et al., 2019; Singh et al., 2023).

#### 3.3.3 SEM and EDX analyses

**FIGURE 2:**
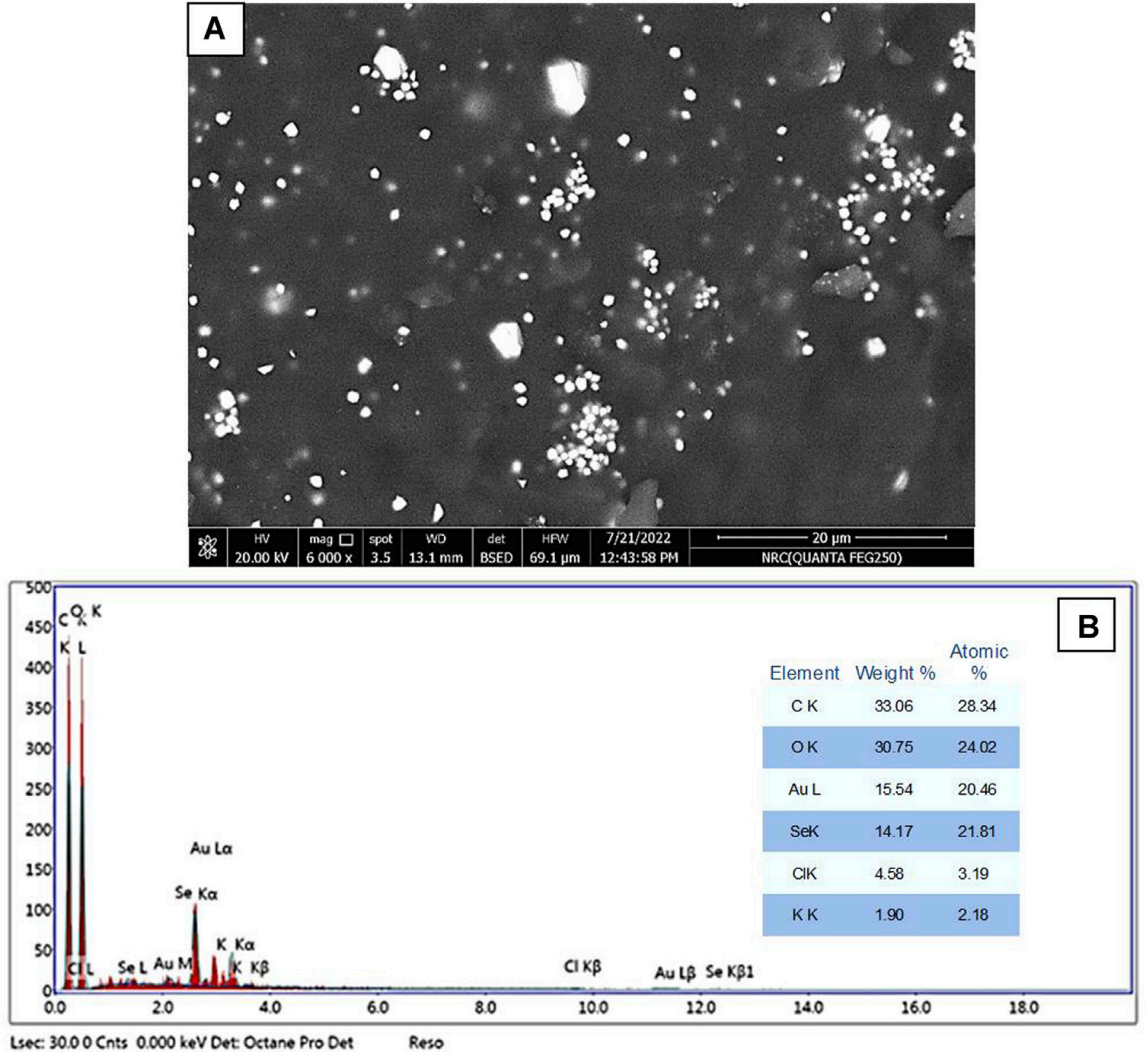
**(A)** SEM image and **(B)** corresponding EDAX spectra of Se–Au BNPs.

The surface morphology and elemental analyses of the produced Se–Au BNPs are shown in Figures 2A, B. Se–Au BNPs, which appear as brilliant particles in *P. indica* extract (Figure 2A), were covered in homogeneous distributions of Se and Au. The elemental structure and chemical validity of the obtained samples were assessed by EDX spectroscopy (Pieła et al., 2020; Elakraa et al., 2022). EDX analysis was conducted to assess the purity and basic structure of the produced Se–Au BNPs, as shown in Figure 2B. The figure illustrates the values of gold at 20.4%, selenium at 21.8%, carbon and oxygen at 28.3% and 24%, respectively, chlorine at 3.1%, and traces of potassium at 2.1%. One of the advantages of green synthesis is the presence of other phytochemical elements from the EDX spectrum that may possibly arise from the *P. indica* leaf extract serving as the capping agent. This is due to the high concentration of capping agents in the reaction medium, the copper grid, or other additives used during the analysis (Negi et al., 2021). The presence of C, O, Cl, and K suggests that the surface of Se–Au BNPs was covered with biomolecules. The results of EDX analysis demonstrated that Se–Au BNPs were successfully synthesized using the *P. indica* leaf extract prepared in this study. Structurally, although the XRD investigation indicated hexagonal crystal formations, it appears that the dominant morphology was rectangular and square-shaped. Silver and selenium make up the manufactured composites, according to Ahmad et al. (2020); the corresponding weight percentages in the intended SeNP–Ag composite are 16% and 84%, respectively. This information was obtained from the EDX spectra. Al-Asfar et al. (2018) used the EDX spectrum to examine the elemental composition of an Ag@Fe nanocomposite. The elemental analysis or chemical characterization of the synthesized SeNPs was performed using the EDX spectrum as described by Hashem et al. (2023).

#### 3.3.4 UV-vis

**FIGURE 3:**
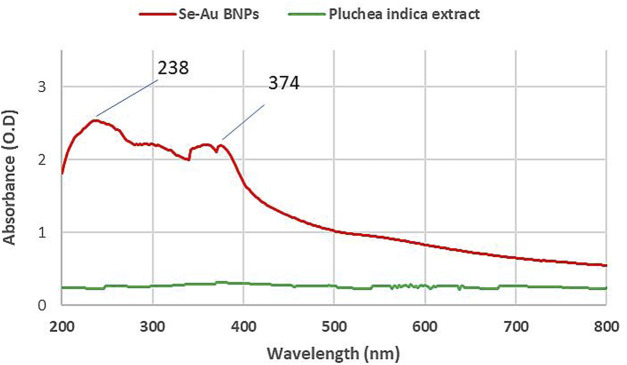
UV-vis absorption spectra of Se–Au BNPs and *P. indica* extract.

To investigate the solution’s nanoparticle content, the UV-vis spectra of the bimetallic Se–Au NPs were also collected (Figure 3). The samples were created by taking the solutions and nanoparticles in their current state and diluting them with deionized water to obtain the curves. The UV-vis spectra of the *P. indica* extract solution did not show any identifiable peaks (Kyriakidou et al., 2021; Qahir et al., 2021). Surface plasmon resonance (SPR) is typically influenced by the intensity dimension, morphological surface, structure, and dielectric characteristics of the generated NPs (Abdelghany et al., 2019). Two peaks in the spectrum, at 238 and 374 nm, were found. However, the peak of selenium nanoparticles at 330 nm suggested that SeNPs had been successfully synthesized (Hashem et al., 2023). The UV-vis analysis of AuNPs at 535 nm demonstrated the effective production of gold nanoparticles. Intriguingly, peaks at 257 and 508 nm were observed in the case of Se–Au–CS, indicating the sequential production of gold and selenium nanoparticles (Zarharan et al., 2023). Ahmad et al. (2020) reported that SPR absorption spectra showed the successful synthesis of Se–Ag hybrid material at 570 nm for selenium nanorods and 450 nm for Ag nanoparticles.

#### 3.3.5 TEM and DLS analyses

**FIGURE 4:**
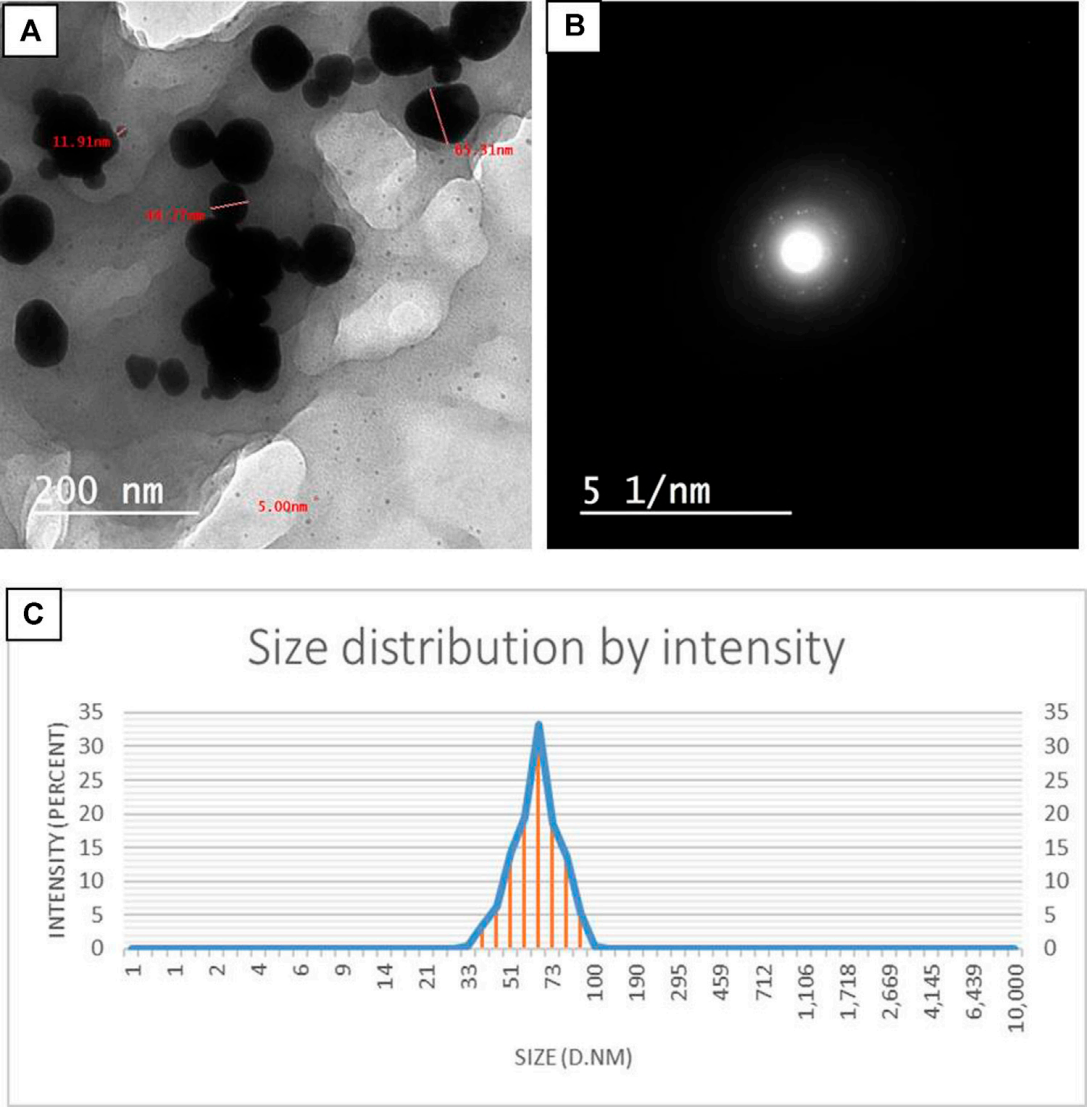
**(A)** TEM images of Se–Au BNPs. **(B)** SAED pattern of Se–Au BNPs. **(C)** Nanoparticle size distribution histograms for Se–Au BNPs.

TEM images of the bimetallic Se–Au nanoparticles were used to analyze their sizes and structures, as shown in Figure 4. The findings suggest that the particles had a mostly spherical morphology, with occasional instances of an oval form. Additionally, the particles were shown to possess a *P. indica* shell, which indicated the aggregation of two or more particles. Figure 4A illustrates the magnitude of Se–Au BNPs; the mean particle diameter was determined to be 44.5 nm, with a range of 5.00–65.3 nm. The SAED recommended looking at the Se–Au BNPs from a polycrystalline perspective (Figure 4B). On the other hand, Zarharan et al. (2023) demonstrated that selenium–gold nanostructures created using chitosan had a particle size range of 100–400 nm, as indicated by TEM images. TEM pictures presented by El-Batal et al. (2020) showed that the produced AgNPs, AuNPs, and bimetallic Ag–AuNPs were shaped in various ways, including spherical and oval shapes. The scale of Ag–AuNPs (Run 4) is also displayed, with a diameter range of 31.65–49.58 nm and a range of 12.58–49.58 nm. Using the DLS method, Ag–AuNPs at Run 4 were found to have an average particle size of 32.58 nm. The UV-visible spectra exhibited two distinct absorption peaks at approximately 250 nm and 360 nm, which can be attributed to the creation of zinc selenide nanoparticles (ZnSeNPs) (Mirzaei et al., 2021). The use of *P. indica* extract as a reducing and capping agent gave the NPs in our study an anisotropic shape and strong stability. Moreover, a comparative study was carried out using the data from dynamic light scattering and transmission electron microscopy. The particle size distribution of the Se–Au BNPs that were biosynthesized with *P. indica* extract was ascertained using the DLS technique. The analysis revealed that the average particle size was 67.4 nm, as shown in Figure 4C. When compared to TEM measurements, DLS size measurements frequently yield better results because they measure the hydrodynamic radius of NPs in the presence of water molecules (solvent), which leads to larger particle sizes of the capped NPs (Jang et al., 2015). On the other hand, TEM examination enables the determination of the actual particle size of the material, excluding the presence of the solvent layer. All of the synthesized NPs had a PDI of 0.29 and were equally spread, according to the most recent DLS data. In contrast, values greater than 0.4 are predicted to result in polydispersity particle diffusion (Saied et al., 2022b). The current data show that the mono-size dispersion of the biosynthesized Se–Au BNPs was modest.

#### 3.3.6 Antibacterial activity

**FIGURE 5:**
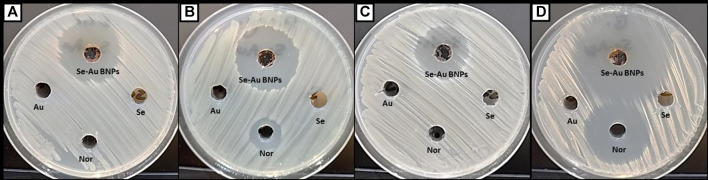
Antibacterial activity of Se–Au BNPs and starting materials toward **(A)** *Escherichia coli*, **(B)** *Pseudomonas aeruginosa*, **(C)** *Staphylococcus aureus*, and **(D)** *Bacillus subtilis*.

Recently, bimetallic nanoparticles have received much attention for their capacity to fight resistant microbes. The results illustrated in Figure 5 revealed that Se–Au BNPs exhibited promising antibacterial activity toward all tested bacterial strains, compared to Na_2_SeO_3_, HAuCl_4_.3H_2_O, and norfloxacin. Specifically, Se–Au BNPs showed potential antibacterial activity toward both *E. coli* and *P. aeruginosa*, where the inhibition zones and MICs were 30.3 ± 1.4 and 31.1 ± 0.8 mm and 31.25 and 15.62 μg/mL, respectively (Table 3). Likewise, Se–Au BNPs exhibited antibacterial activity toward Gram-positive bacteria, among which *B. subtilis* was the most sensitive bacterium, with an inhibition zone and MIC of 45.6 ± 1.0 mm and 3.9 μg/mL, respectively. In contrast, *Staphylococcus aureus* was the least sensitive; its inhibition zone and MIC were 26.1 ± 1.1 mm and 31.25 μg/mL, respectively. Nor, as a standard antibiotic, showed less antibacterial activity than Se–Au BNPs, which exhibited activity against *P. aeruginosa* and *B. subtilis*. On the other hand, all the starting materials (Na_2_SeO_3_, HAuCl_4_⋅3H_2_O, and leaf extract of *P. indica*) showed no activity toward any of the bacterial strains tested. In a recent investigation, mono- and bimetallic silver–gold nanoparticles were successfully synthesized using starch as a reducing and stabilizing agent. These nanoparticles exhibited noteworthy antibacterial properties against MDR *E. coli* and methicillin-resistant *Staphylococcus aureus* (MRSA) strains (Lomelí-Marroquín et al., 2019). Furthermore, bimetallic silver–selenium nanoparticles were biosynthesized in a green manner using *Orobanche aegyptiaca* extract; these nanoparticles showed superior antimicrobial and antibiofilm activity against *Staphylococcus aureus*, *P. aeruginosa*, and *Candida albicans* (Mostafa et al., 2023). Furthermore, pomegranate peel extract was used for the biosynthesis of bimetallic silver/zinc oxide nanoparticles that exhibited antimicrobial activity against *E. coli*, *P. aeruginosa*, *B. subtilis*, *Staphylococcus aureus*, *Escherichia faecalis*, *Candida albicans, Cryptococcus neoformans, Aspergillus fumigatus*, and *A. brasiliensis* (Hashem and El-Sayyad, 2023). Investigations have also been conducted of the antifungal activity of Picoa–AgNPs against three pathogenic fungi, namely, *Pythium* sp*., Aspergillus flavus*, and *Aspergillus niger*, which can cause severe damage to health and agriculture (Owaid et al., 2022). The antibacterial mechanism of bimetallic Se–AuNPs may be attributed to the combined effects of SeNPs and AuNPs. These nanoparticles can adhere to bacterial cells by charge attraction, resulting in alterations to the bacterial membrane. This, in turn, leads to changes in microbial permeability owing to the breakdown of the membrane. Moreover, nanoparticles can alter enzymes and proteins, cause oxidative stress and production of reactive oxygen species (ROS), and interfere with the electron transport chain (Baptista et al., 2018).

**TABLE 3:** Inhibition zones and MICs of bimetallic Se–Au NPs and starting materials.

Bacterial strain	Se–Au BNPs		Se (IZ)	Au (IZ)	Nor	
	IZ/mm	MIC (µg/mL)			IZ/mm	MIC (µg/mL)
*E. coli*	30.3 ± 1.4	31.25	0.0	0.0	0.0	ND
*P. aeruginosa*	31.1 ± 0.8	15.62	0.0	0.0	17.5 ± 0.7	125
*S. aureus*	26.1 ± 1.1	31.25	0.0	0.0	0.0	ND
*B. subtilis*	45.6 ± 1.0	3.9	0.0	0.0	34.7 ± 1.0	31.25

IZ, inhibition zone.

#### 3.3.7 Anticancer activity

**FIGURE 6:**
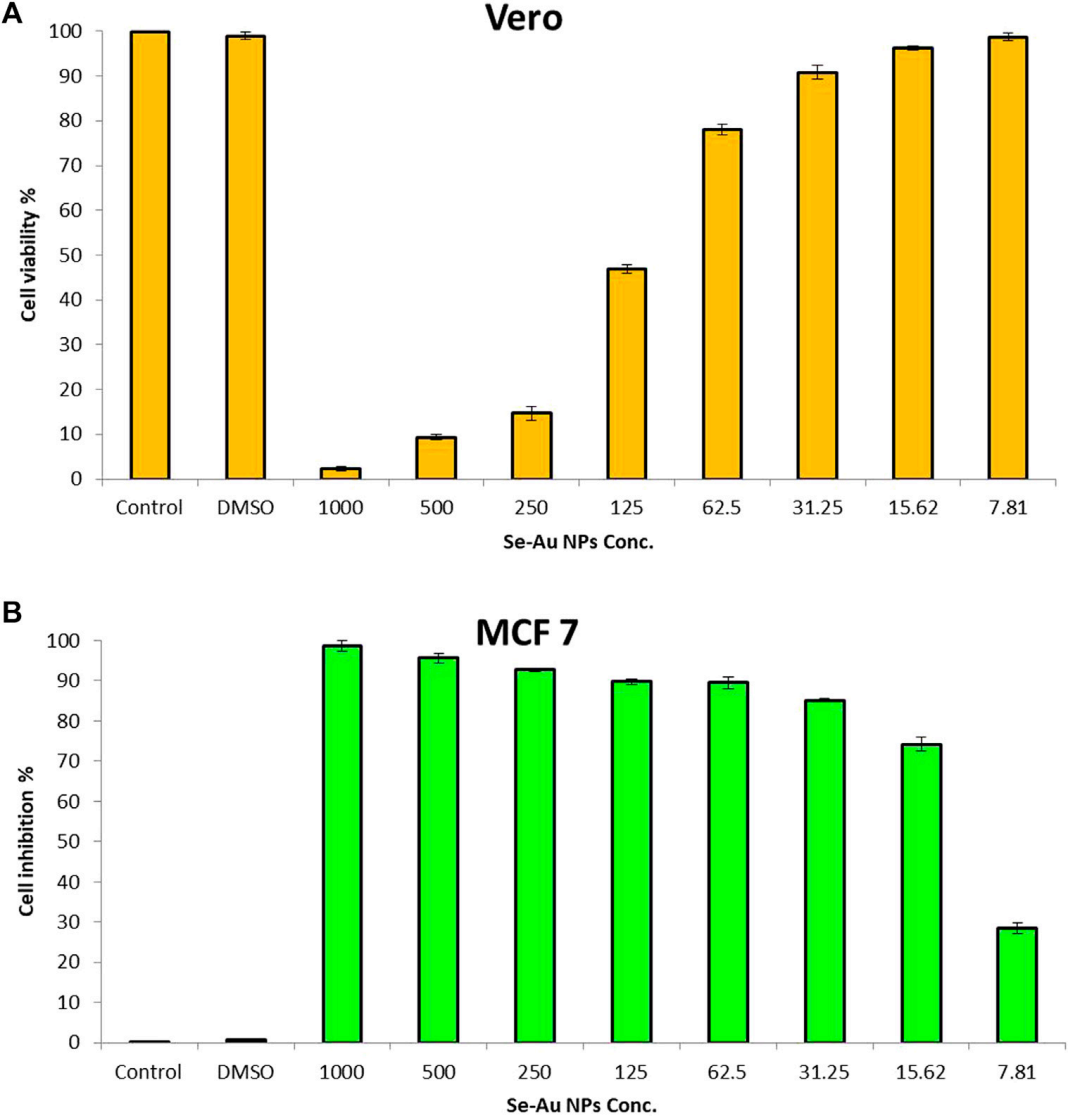
Cytotoxicity of Se–Au BNPs toward **(A)** the Wi38 normal cell line and **(B)** the MCF7 cancerous cell line. Control = Wi38/MCF8 control; DMSO = dimethyl sulfoxide.

In general, the initial stage in assessing the biosafety of substances involves the examination of their cytotoxicity. The cytotoxicity of Se–Au BNPs was tested using the Wi 38 normal cell line in this work, as illustrated in Figure 6. The results revealed that the cell viability of the Wi 38 cell line at concentrations of 7.81, 15.62, 31.25, and 62.5 μg/mL was 98.9, 96.4, 90.9%, and 78.1%, respectively. Furthermore, the results confirmed that the biosynthesized bimetallic Se–Au BNPs are safe for use, with an IC_50_ of 116.8 μg/mL. According to Ioset et al. (2009), if the IC_50_ is ≥90 μg/mL, the compound or material is classified as non-toxic.

The anticancer activity of Se–Au BNPs was evaluated using the MCF7 cancerous cell line, as shown in Figure 6. The results indicated that bimetallic Se–Ag NPs showed anticancer activity, with an IC_50_ of 13.77 μg/mL. According to the cytotoxicity of Se–Au BNPs toward the Wi 38 normal cell line, safe concentrations are 62.5, 31.25, 15.62, and 7.81 μg/mL, which exhibited anticancer activity of 89.2, 85, 74.3, and 28.8%, respectively. The anticancer activity of nanoparticles is commonly associated with many processes, including the induction of ROS-mediated apoptosis. This mechanism is widely recognized as a key contributor to the anticancer effects of nanoparticles, as it leads to detrimental effects on the cell membrane, mitochondrial malfunction, and enzymatic and protein oxidation, as well as DNA breakage (Kim et al., 2020) and the modulation of apoptotic regulatory proteins, leading to the initiation or suppression of programmed cell death (Grueso et al., 2019). Lomelí-Marroquín et al. (2019) reported that bimetallic Ag–Au NPs showed anticancer activity against human melanoma cells. Similarly, Nieto-Argüello et al. (2022) confirmed that the use of bimetallic Ag–Au nanoparticles exhibits an anticancer impact on melanoma cells. This effect may be related to excessive ROS generated within the surrounding medium.

#### 3.4 Limitations of the study

In our future studies, the Se–Au BNPs biosynthesized in the current study will be evaluated in terms of activity toward multiple normal and cancerous cell lines *in vitro*. Furthermore, *in vivo* cytotoxicity will be assessed in animal models to confirm safety in normal cells, as well as effectiveness in cancerous cells.

## 4 Conclusion

In the current study, the phytochemicals present in *P. indica* leaf extract were used for the first time to reduce, cap, and stabilize Se–Au BNPs. The phytochemical analysis illustrated the presence of significant quantities of phenolics, flavonoids, and tannins, which play a principal role in the green and ecofriendly biosynthesis of bimetallic nanoparticles. As-formed Se–Au BNPs were characterized using UV-vis, XRD, TEM, DLS, SEM-EDX, and FTIR analyses. The maximum UV-vis spectra for the biosynthesized Se–Au BNPs were observed at two peaks (238 and 374 nm). Additionally, a crystalline nature, with well-dispersed, spherical-shaped nanoparticles with an average size of 44.5 nm, was detected in XRD, DLS, and TEM analyses. The distinct peaks observed in the FTIR analysis correspond to different functional groups present in *P. indica* extract, which play a crucial role in stabilizing and reducing Se–Au BNPs. The biogenic Se–Au BNPs showed antimicrobial activities against the pathogens *Staphylococcus aureus*, *B. subtilis*, *P. aeruginosa*, and *E. coli*, with a varied zone of inhibition at safe concentrations, according to cytotoxicity results in a normal cell line. The data showed that the Se–Au BNPs exhibited potential antibacterial activity toward both *E. coli* and *P. aeruginosa*, with inhibition zones and MICs of 30.3 ± 1.4 and 31.1 ± 0.8 mm, and 31.25 and 15.62 μg/mL, respectively. Similarly, Se–Au BNPs exhibited antibacterial activity toward Gram-positive bacteria, among which *B. subtilis* was the most sensitive bacterial strain, with an inhibition zone and MIC of 45.6 ± 1.0 and 3.9 μg/mL, respectively. *Staphylococcus aureus* was the least sensitive bacterium, with an inhibition zone and MIC of 26.1 ± 1.1 mm and 31.25 μg/mL, respectively. Moreover, Se–Au BNPs showed promising anticancer activity toward the MC7 cancerous cell line, with an IC_50_ of 13.77 μg/mL. Ultimately, it will be possible to use the Se–Au BNPs biosynthesized in this study in biomedical fields after extensive *in vitro* and *in vivo* studies.

## Data Availability

The raw data supporting the conclusions of this article will be made available by the authors, without undue reservation.
